# Supplementary material for: GDC Cohort Copilot: an AI copilot for curating cohorts from the genomic data commons
Source: Bioinform Adv. 2025 Nov 18;5(1):vbaf295. doi: 10.1093/bioadv/vbaf295 (PMC12677940; doi:10.1093/bioadv/vbaf295)
Supplement: vbaf295_Supplementary_Data [file vbaf295_supplementary_data.zip › 24-Nov-2025_113334_GDC_Cohort_Copilot___Bioinformatics_Advances_Format__Supplemental.pdf]

Supplementary Materials

Random Cohort Generation

We augment our existing user-derived cohort data by creating synthetic cohort filter JSONs through a two-step stochastic process. To create a new JSON filter, we first select the number of fields, n, to include by sampling a nonzero integer value from a Chi-Square distribution with 6 degrees of freedom, using rejection-sampling to ensure i.i.d. samples. Next, we randomly select n distinct fields from the core set of filter properties. For fields with numerical ranges, we randomly select a filter operator (one of $\leq$, $<$, $\geq$, $>$) and a corresponding numerical value within the field's allowable range. Conversely, all non-numerical fields use an "in" operator with a list of values. To create this list of filtering values for a given field, we first randomly select the number of values, m, with a minimum of 1 and a maximum of 5. The m values are then randomly selected from the list of possible values for the given field. We construct each filter as a JSON object and validate it against the GDC cohort schema to ensure correctness. Synthetic filter uniqueness is strictly enforced by computing an MD5 hash digest of the sorted JSON representation and rejecting all duplicates. We iteratively generate filters until the target number of unique, valid, synthetic cohort filters are generated.

Model Training Details

We describe here our model training details for our experiments in developing GDC Cohort LLM. All models were trained using HuggingFace Trainer on single NVIDIA A100 80GB GPU. For all experiments, we use a linear learning rate schedule with an initial learning rate of 5e-5, 8 gradient accumulation steps, and a maximum sequence length of 1,024. For training the LoRA adapter for Mistral, we target the following layers: `q_proj, k_proj, v_proj, o_proj, gate_proj, down_proj, up_proj`. Specific hyperparameters for each experiment are further detailed in Supplementary Table 6.

Comparison to GPT-4o

To compare our trained GDC Cohort LLM against an expensive, closed source alternative using OpenAI's GPT-4o, we adopt the following prompting strategy. We use the OpenAI python library v1.84.0 to send requests to the OpenAI API. We use OpenAI's Structured Outputs by specifying our Pydantic data model (Section 2.2) as the response format to chat completions. This same data model and corresponding JSON schema are also used by our locally served model. We pin the model version to `gpt-4o-2024-08-06`, set temperature to 0, set a fixed generation seed, and specify a maximum of 1,024 completion tokens. We use the prompt presented in Supplementary Table 7. To provide GPT-4o with the set of all possible filter fields and their values without model tuning, we input in the prompt the entire field-value mapping for the core set of filter properties. This mapping is approximately 15,000 tokens long and thus, regardless of performance metrics, this method is infeasible for scalable inference as the token cost rapidly compounds. At the time of our experiments, the cost of our prompt to GPT-4o was approximately $0.04.

Reverse Translation

In our dataset of real user-derived cohort filters (originally, assembled using the GDC Cohort Builder), the cohort filters are JSON strings. As we aim to train an LLM to generate these filters using natural language, we develop a reverse-translation method to prompt an LLM to generate a JSON filter's corresponding natural language description. Specifically, we use `mistralai/Mistral-7B-Instruct-v0.3` for this task without any additional fine-tuning. We serve the model using vLLM on a single NVIDIA A100 80GB GPU and generate natural language queries using a set seed, temperature 0, and a maximum of 4,096 completion tokens. We use in-context learning to prompt the model with 2 examples of our task; our precise prompt for reverse-translation is provided in Supplementary Table 8. We use this same process to generate natural language queries for our synthetic data samples (Section A.1). Additionally, we use this method in our evaluation of semantic similarity between two cohort filters (Section 2.3.3).

Supplementary Tables

Table 4. GPT-2 significantly outperforms ($p < 0.05$), or is not meaningfully worse than, BART and Mistral as foundations for GDC Cohort LLM, when trained over user-derived cohort filters. Significantly better results are **bolded**.

Model Type	TPR	IoU	Exact	BERT
GPT-2	**0.365**	**0.331**	**0.221**	0.819
BART	0.117	0.078	0.028	0.735
Mistral	0.124	0.117	0.092	**0.835**

Table 5. Training over synthetic data mixtures significantly improves GDC Cohort LLM performance ($p < 0.05$). Significantly better results are **bolded**.

Data Mixture	TPR	IoU	Exact	BERT
Real	0.365	0.331	0.221	0.819
Real + 100K Synthetic	0.783	0.748	0.607	0.902
Real + 1M Synthetic	**0.855**	**0.832**	**0.702**	**0.919**

Table 6. Model training hyperparameters for GDC Cohort LLM experiments. All models were trained on a single NVIDIA A100 80GB GPU.

Model	Trainable Params	Train Epochs	Batch Size	Precision	Specifics
Mistral	11M	1	64	bf16	LoRA(r=4, α=16, dropout=0.1)
BART	139M	3	32	bf16	warmup steps=0
GPT-2	124M	10	32	fp16	warmup steps=500
GPT-2-100K	124M	10	32	fp16	warmup steps=500
GPT-2-1M	124M	10	32	fp16	warmup steps=500

Table 7. Prompt to derive cohort filters using OpenAI's GPT-4o. The field-value list is populated using a predefined mapping for all core set filter properties. This list consumes approximately 15,000 tokens. Cohort filters are generated using structured decoding.

Line	Role	Message
1	System	Construct NCI GDC cohort filters based on the input cohort description using the given list of possible fields and values.
2	User	Here is the list of possible fields and values:<br><br>[Field-Value List]<br><br>Use the above properties to construct a NCI GDC cohort filter for the following cohort description:<br>[Query]

Table 8. Prompt to reverse-translate natural language descriptions of input cohort filters using an LLM. `{Filter}` is replaced by the target cohort filter to reverse-translate.

```
Given the following examples of dict and sentence pairs, generate the
sentence that describes a new dict between <<>>.
Use the 'field' and it's corresponding 'value' information to correctly
identify the different categories.
Examples:

Example 1:
Dict :
{'op': 'and',
 'content': [{'op': 'in',
   'content': {'field': 'cases.project.program.name', 'value': ['TARGET']}},
  {'op': 'in',
   'content': {'field': 'cases.project.project_id',
    'value': ['TARGET-ALL-P1',
     'TARGET-ALL-P2',
     'TARGET-ALL-P3',
     'TARGET-AML']}},
  {'op': 'in',
   'content': {'field': 'cases.diagnoses.site_of_resection_or_biopsy',
    'value': ['bone marrow']}},
  {'op': 'in',
   'content': {'field': 'cases.samples.tissue_type', 'value': ['tumor']}},
  {'op': 'in',
   'content': {'field': 'cases.samples.tumor_code',
    'value': ['acute lymphoblastic leukemia (all)']}}]}
Sentence :
acute lymphoblastic leukemia tumor code for bone marrow tumors that belong to
TARGET-ALL-P1, TARGET-ALL-P2, TARGET-ALL-P3, TARGET-AML projects. |<eos>|

Example 2:
Dict:
{'op': 'and',
 'content': [{'op': 'in',
   'content': {'field': 'cases.project.program.name', 'value': ['CGCI']}},
  {'op': 'in',
   'content': {'field': 'cases.project.project_id', 'value': ['CGCI-BLGSP']}},
  {'op': 'in',
   'content': {'field': 'cases.diagnoses.tissue_or_organ_of_origin',
    'value': ['hematopoietic system, nos']}},
  {'op': 'in',
   'content': {'field': 'cases.samples.preservation_method',
    'value': ['ffpe']}}]}
Sentence:
ffpe samples for hematopoietic system, nos that belong to the CGCI-BLGSP
project. |<eos>|


<<{Filter}>>


Sentence:
```
